# Predictors of Chronic Hepatitis C Evolution in HIV Co-Infected Patients From Romania

**DOI:** 10.5812/hepatmon.8611

**Published:** 2013-02-28

**Authors:** Camelia Sultana, Simona Manuela Erscoiu, Camelia Grancea, Emanoil Ceausu, Simona Ruta

**Affiliations:** 1 Department of Virology, Carol Davila University of Medicine and Pharmacy, Bucharest, Romania; 2 Emergent Disease Department, Stefan S. Nicolau Institute of Virology, Bucharest, Romania; 3 Infectious Disease Department, Carol Davila University of Medicine and Pharmacy, Bucharest, Romania; 4 Infectious Disease Department, Victor Babes Infectious and Tropical Diseases Hospital, Bucharest, Romania

**Keywords:** Coinfection, Romania, Biological Markers

## Abstract

**Background:**

Due to a recent alarming increase in the number of HIV-HCV co-infected patients in Romania.

**Objectives:**

A cross sectional study was conducted to assess the baseline predictors of liver disease evolution.

**Patients and Methods:**

83 HIV-HCV co-infected patients, untreated for HCV infection, were evaluated for viral replication, liver fibrosis (estimated by a noninvasive marker - FIB4), and plasma levels of IP-10 (interferon-gamma inducible protein 10) - a cytokine associated with an unfavorable outcome of HCV infection.

**Results:**

The median value for HCV viral load was high (6.3 log_10_ IU/mL), 98.8% of the patients were infected with HCV genotype 1. Although 53% of the patients received antiretroviral therapy (cART), only 31.8% of these achieved undetectable HIV levels. HCV viral load was significantly higher in patients with AIDS (6.4 vs. 6.1 log_10_IU/mL; P = 0.04), and in those naïve for cART (6.5 vs. 5.9 log_10_ IU/mL; P = 0.04). Severe fibrosis was directly correlated with immunosupression (56% vs. 17.4%, P = 0.03), HCV replication (6.1 vs. 4.9 log_10_IU/mL P = 0.008), and IP-10 median values (312 vs. 139 pg/ml, P=0.008). A serum IP-10 level higher than 400 pg/mL was significantly associated with FIB-4 median values (4.09 vs. 1.7, P = 0.004), HCV viral load (6.4 vs. 6.1 log_10_ IU/mL, P = 0.02) and ALT level (206.8 vs. 112.4 IU/L, P = 0.05).

**Conclusions:**

An important part of the HIV-HCV co-infected patients had negative baseline predictors for the evolution of HCV infection; their therapeutical management must be conducted with special attention towards adherence and potential overlapping drug toxicities. High concentrations of plasma IP-10 are reliable markers for the severity of liver disease.

## 1. Background

During the last two decades human immunodeficiency virus (HIV) and hepatitis C virus (HCV) have generated intertwined epidemics; worldwide around 30% of the 34 million HIV-infected individuals being co-infected with HCV, with the highest rates in injection drug users (1). End stage- liver disease caused by chronic HCV infection is nowadays the leading cause of morbidity and mortality in HIV-infected patients who show increased proportions of cirrhosis compared to the HCV mono-infected ones, as well as poorer responses to the standard combined pegylated interferon (PEG-IFN) and ribavirin therapy (2, 3). Accelerated hepatic injuries in HIV-HCV co-infected patients are attributed both to HIV-driven immune activation and cytotoxic CD8 T cells accumulation in the liver, as well as to HIV direct replication in hepatocytes and hepatic stellate cells and inducement of apoptosis (4). The HCV epidemics from Central and Eastern Europe has been fueled lately by the large population of HIV infected individuals. Romania had experienced a different epidemiological situation, most of the HIV cases (10,903 subjects in 2012) were derived from the nosocomial pediatric epidemic of the early 90s (5), with infrequent co-infection with HCV (6). After 2000, a slow but constant increase was reported in the number of adult HIV cases, mostly infected heterosexually, and recently, an alarming rise in the number of HCV infections in intravenous drug users has been reported (IDUs) (7). Consequently, one third of the total cumulative number of HIV-HCV co-infections reported between 1989-2011 were diagnosed in 2011 only (102/332 cases) (8).

## 2. Objectives

The current cross-sectional study to assess the baseline virological and biochemical predictors of the evolution of HCV-related liver disease was conducted due to the recent abrupt increase in the number of the HIV-HCV co-infected patients, and the scarcity of data available on this issue in Romania.

## 3. Patients and Methods 

### 3.1. Patients

Socio-demographic, clinical and biochemical data were collected from the medical records of 83 HIV-HCV co-infected patients admitted, mainly for opportunistic infections, in one of the main clinics of infectious diseases in Bucharest, during 2009–2011. Informed consent was obtained from all patients, and the study was approved by the Bioethics Committee of the Stefan S. Nicolau Institute of Virology.

### 3.2. Virological Monitoring

HCV viral load (HCV VL) was tested by RT-PCR (CobasAmplicor HCV Monitor, vers 2.0, Roche; linear range between 600 -700,000 IU/mL, lower detection limit 600 IU/mL). HCV genotyping was performed using a commercial Line Probe Assay (LINEAR ARRAY Hepatitis C Virus Genotyping Test, Roche) which discriminates between the 6 major HCV genotypes. For HIV infection follow-up, plasma HIV viral load (HIV VL) was measured by real-time PCR (COBAS Taqman HIV-1 Test, Roche; linear range between 48- 10,000,000 copies HIV RNA/mL, lower detection limit 48 copies/mL); immunological status was assessed as absolute number of CD4 cells/mm3, using Multitest CD3 FITC/CD8 PE/ CD45 PerCP/CD4 APC Reagent, Becton-Dickinson. Liver fibrosis was evaluated with a composite biochemical index - FIB-4, and was calculated by Sterling's formula (9): Age [years] × AST [IU/L] /platelets [× 10^9^/L] × (ALT1/2[IU/L]). FIB-4 values less than 1.45 show minimal fibrosis, while FIB-4 > 3.25 indicate significant fibrosis. For ALT level, the upper limit of normal (ULN) was 30 IU/L for men, and 19 IU/L for women, elevated values were defined according to ACTG criteria (10), from grade 1 (1.2–2.5 × ULN) to grade 4 (> 10 × ULN). Detection of plasma levels of CXCL10/IP-10 (interferon-gamma inducible protein 10) was conducted using a quantitative sandwich immunoassay (Quantikine Human CXCL10/IP-10, R&DSystems), with minimum detectable dose ranging from 0.41 - 4.46 pg/ and a linear range between 0 - 500 pg/mL. GraphPadInStat 3 Program was employed to analyze the data, with Fisher Exact test (for contingency tables), ANOVA and unpaired t test (for mean values); a P value < 0.05 was considered statistically significant.

## 4. Results 

### 4.1. Characteristics of Study Subjects

The mean age of the co-infected patients was 35.5 ± 11 years, 74.7% were male, 79.5% were from urban areas, with a medium level of education (61.5% graduated from secondary school), and 68.7% were unmarried or divorced. The mean duration of infection was 6.5 ± 4.9 years for HIV and 6.1 ± 4.7 years for HCV. In 65.1% of cases both infections were detected concomitantly; only in 7 patients (8.4%), all IDUs, HCV infection was the first diagnosed. Most of the subjects had multiple risk factors for the acquisition of both infections (42.2%- IDU; 43.4%- multiple heterosexual partners; 18.1%- repeated parenteral treatments and surgical procedures, 16.9%- transfusions before 1990).Most of the patients were in advanced stages of HIV infection: 34.9% in stage B3 and 49.4% in stage C3, according to the CDC classification. The majority presented high HIV viral loads (median 5.2 log_10_ copies/mL), despite the fact that 53% were receiving combined antiretroviral therapy (cART); 68.1% of these with more than one therapeutic regimen overtime. All patients were naïve for HCV treatment at the time of testing. Only 14 patients (31.8% of the treated ones) had undetectable HIV viremia. The median CD4 cell number was 300 cells/mm^3^; 23 patients (27.7%) had severe immunosuppression (less than 200 CD4 cells/mm^3^). Table 1 presents the patients’ characteristics, according to the degree of immunosuppression.

**Table 1:** Impact of HIV-Related Immunosuppression on the HCV-Induced Liver Disease

	Total Patients, No. (n=83) [Table-fn fn1171]	Patients With CD4 < 200 a (n=23)	Patients With CD4 200-500 (n=37)	Patients With CD4 > 500 (n=23)	P value b
**Median HIV VL, Log_10_ copies /mL**	5.2	5.5	5.2	4.3	0.02
**cART treated, No. (%)**	44 (53)	16 (69.6)	22 (59.5)	6 (26.1)	0.007
**Median HCV VL, Log_10_ IU/mL**	6.3	6.3	6.3	6.2	0.94
**Median FIB4 index**	2.14	3.34	2.14	1.11	0.03
**Patients with FIB4 > 3.25, No. (%)**	29 (34.9)	13 (56.5)	12 (32.4)	4 (17.4)	0.03
**Mean ALT, IU/L**	131.5	133.2	148.6	91.4	0.45

Abbreviations: ALT, Alanine transaminase; cART, combined antiretroviral therapy; HCV, hepatitis C virus; HIV, human immunodeficiency virus; VL, viral load

^a^CD4 count is expressed in cells/mm^3^

^b^P value < 0.05 (for CD4 < 200 vs. CD4 > 500) was considered statistically significant

### 4.2. HCV Viral Load (VL) and Genotypes

The median baseline value for HCV VL was high (6.3 log_10_ IU/mL), 44 patients (53%) had values higher than 600,000 IU/mL, while only 12 (14.5%) patients had undetectable HCV RNA. HCV VL was significantly higher in patients with AIDS (6.4 vs. 6.1 and 6 log_10_ IU/mL; for those in clinical stages A and B, respectively; P = 0.04), and in patients naïve for cART (6.5 vs. 5.9 log_10_IU/mL for treated patients; P = 0.04). No direct statistic correlation was established between the values of HCV and HIV viral loads. All but one of the HIV-HCV co-infected patients (98.8%) were infected with HCV genotype 1; the only exception was a 40 -year- old heroin drug-user, diagnosed in 2009, infected with HCV genotype 4 and treatment-naïve for both infections.

### 4.3. Assessment of Liver Disease

**Figure 1:**
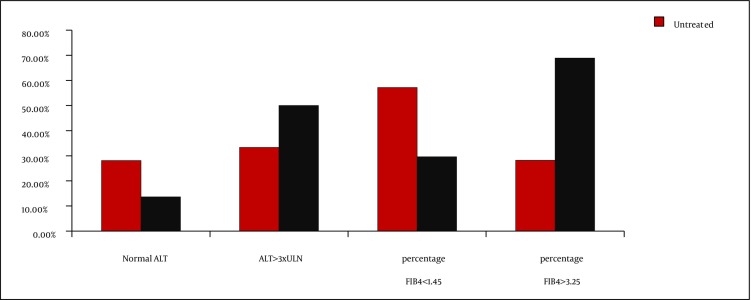
Correlation Between cART Treatment, FIB-4 Index and Plasma ALT Levels

Advanced liver fibrosis, as defined by FIB-4 values > 3.25, was present in 34.9% of the patients under study, while 32.5% had minimal hepatic damages (FIB-4 lower than 1.45). Increased FIB-4 values were associated with high HCV viral loads (6.1 vs. 4.9 log_10_ IU/mL; P = 0.008) and immunosuppression (high median value of FIB4 index and increased percentage of patients with FIB4 values > 3.25 [Table 1]). Significant decrease of CD4 cell number in patients with advanced liver fibrosis were also detected (432 vs. 232 cells/mm^3^; P = 0.004 [Table 2]). A significantly higher percentage of cART treated patients had advanced liver fibrosis (68.9% vs. 29.6%, with FIB-4 values > 3.25 P = 0.007) (Table 2, Figure 1). cART was associated with a decrease in the mean value of HCV viral load, but not with achievement of undetectable HCV level. Remarkably, 41% of the study subjects had normal ALT levels, while high grades of ALT elevation were present in only 28.9% of the cases, especially in patients treated with cART-Figure 1, but without significant correlation with the degree of the immunosuppression, HIV viral load, and FIB-4 index median values.

**Table 2:** Association Between Liver Fibrosis and Virological Markers

Parameter	Patients with minimal/none liver fibrosis FIB-4 < 1.45 (n=27) [Table-fn fn1174]	Patients with advanced liver fibrosis FIB-4 > 3.25 (n=29)	P value a
**Median HCV VL, log_10_ IU/mL **	4.9	6.1	0.008
**IP-10 Median value, pg/mL **	139	312	0.008
**Median HIV VL, Log_10_ copies /mL**	5	5.1	0.26
**Treated with cART, No. (%)**	8 (29.6%)	20 (68.9%)	0.007
**Median CD4 cell number, cells/mm^3^**	432	232	0.004

Abbreviations: cART, combined antiretroviral therapy; HCV, hepatitis C virus; HIV, human immunodeficiency virus; VL, Viral Load

^a^P value < 0.05 is considered statistically significant

### 4.4. Correlation Between IP-10 Levels and the Parameters of HCV Infection

The median IP-10 level in the subjects under study was 189 pg/mL (range 44 - 754).Only 6% of the patients under study had IP-10 levels lower than 96 pg/mL (the mean value for healthy volunteers). All these patients were treated for HIV infection, had low HCV and HIV viral loads, and low ALT levels and FIB-4 index values. Minimal liver fibrosis was more frequently encountered in patients with lower levels of IP-10 (37.9% vs. 12%, P = 0.02; 95%CI = 1.052-1.767). Overall, an elevated concentration of plasma IP-10 (> 400 pg/ml) was correlated with a significant fibrosis level (mean FIB-4 values 4.09 vs. 1.7, P = 0.004) and increased ALT levels (206.8 vs. 112.4 IU/L, P = 0.05) [Table 3]. Median HCV and HIV viral loads, as well as CD4 cell count values were significantly higher in patients with IP-10 levels greater than 400 pg/mL.

**Table 3:** Correlation Between Plasma IP-10 Levels and HCV Liver Disease Parameters

	Patients with IP-10 levels < 400, pg/mL (n = 58)[Table-fn fn1175]	Patients with IP-10 levels > 400, pg/mL (n = 25)	P value a
**Median HCV VL, log_10_ IU/mL**	6.1	6.4	0.02
**Patients with FIB-4 index < 1.45, No. (%)**	22 (37.9%)	3 (12%)	0.02
**Median FIB-4 Index **	1.7	4.09	0.004
**Mean ALT, IU/L**	112.4	206.8	0.05
**Median HIV VL, log_10_ copies/mL**	3.9	5.2	0.05
**Median CD4 cell number, cells/mm^3^**	141.5	550	< 0.001

Abbreviations: ALT, Alanine transaminase; HCV, hepatitis C virus; HIV, human immunodeficiency virus; VL, viral load

^a^P value < 0.05 is considered statistically significant

## 5. Discussion

Several baseline predictive factors for the natural and on treatment HCV infection evolution were reported for both HCV mono-infected and HIV co-infected patients: HCV viral load, HCV genotype, the degree of liver fibrosis together with a host genetic factor, and the IL28B polymorphism (11). Almost all of the co-infected patients from our cohort were infected with HCV genotype 1 - the most hard-to-treat with the standard PEG-IFN/ribavirin combination; more than half had HCV viral load higher than 600 000 IU/ml and more than a third presented advanced liver fibrosis, with high FIB-4 values. FIB-4, a non-invasive biochemical index, is an accurate marker for advanced liver fibrosis in both HCV infection and HIV-HCV co-infection (12, 13), and a realistic alternative to liver biopsy - a method which, despite its accuracy, is associated with potential severe complications, sampling errors and low patients’ compliance. In addition, the degree of fibrosis was directly correlated with immunosuppression, a fact that might impact the decision to treat, as the majority of the safety/efficacy studies in co-infected patients are based on observations of the ones with more than 200 CD4 cells/mm3. All these baseline characteristics of the co-infected patients are in accordance with other studies suggesting that the rate of HCV spontaneous clearance, the extent of HCV replication and the degree of hepatic fibrosis are negatively influenced by HIV infection (1, 4, 14). Although cART can provide a significant reduction in the HCV induced necro inflammatory activity in co-infected patients with relatively preserved immune status, it can also increase fibrosis through cumulative hepatotoxicity or immune restoration (4); in the current study, treated patients tended to have significant fibrosis more frequently. As long as the majority of these patients have low levels of CD4, it is unlikely that cART-induced immune restoration triggers the fibrosis. It is worth mentioning that adherence to ART was suboptimal in our cohort, accounting for the high rate of HIV treatment failures, with important percentages of patients with elevated HIV viral loads and moderate/severe immunosuppression. It cannot be overlooked that an increase in the rate of fibrosis might be attributed to the cumulative drug-induced hepatotoxicities during cART (15), especially taking into account the higher percentage of treated patients with high grade elevation of ALT. While in the entire cohort more than 40% of subjects had normal serum ALT levels, which does not exclude the absence of significant liver damage; cirrhosis have been diagnosed by liver biopsy in 12-14% of co-infected subjects with normal ALT (16). A combined therapy for HIV and HCV infection, although a priority, might prove difficult to conduct under these conditions, because of metabolic complications and synergistic toxicity profiles; nevertheless, the benefits of a well-tailored treatment outweigh the potential risks. Recent international guidelines (17) recommend treatment initiation, even in co-infected patients with cirrhosis, if certain antiretroviral drugs (in particular didanosine and stavudine, which cause mitochondrial toxicity and microvesicularsteatosis) are avoided, and if the regimen is adjusted according to drug interactions and overlapping toxicities. Generally, only a small proportion of HIV-HCV co-infected patients can be treated successfully for HCV infection with standard therapy (PEG-IFN alpha plus weight-based doses of ribavirin) and the regimen is poorly tolerated. The reported rates of sustained virological response are substantially lower than in HCV mono-infected patients, ranging from 27% to 40% (3, 18). The poor therapeutic response of the co-infected individuals can be attributed either to the HIV- induced immune activation, or to the HCV-induced immunoregulatory and pro-inflammatory pathways, with an aberrant type-I IFN response (19). However, retrospective repeated liver biopsy analysis had shown that HCV treatment can stop development of fibrosis and even induce its regression (20). In addition, it can also have a positive impact on the HIV disease progression, as long as several recent reports suggest that HCV augments the development of AIDS-defining conditions (14) and diminishes the immune recovery (21). Moreover, HIV-HCV co-infected patients who fail to achieve a sustained virological response have significantly higher risk of HIV progression and increased mortality rates, which is not related to liver disease or to AIDS-related conditions (22). It is essential to highlight the fact that the Romanian national waiting-list for HCV therapy exceeds 5,000 patients (23), the vast majority being HCV mono-infected, with advanced liver disease and predictable rapid evolution to cirrhosis. The addition of the highly vulnerable group of HIV-HCV co-infected patients will inflict important economic consequences; the number of HCV-treated patients is relatively low in Romania, mainly due to the lack of funds (between 2002-2009 not more than 4.1% of the total reported number of those HCV infected received treatment) (23). Consequently, the correct monitoring and adequate selection of the best candidates for standard or future triple therapy (including protease inhibitors) in this increasing population of HIV-HCV co-infected patients, is of outmost importance (24).An important finding of the current study is the correlation between the plasma level of IP-10 and the degree of liver fibrosis in HIV-HCV co-infected patients. IP-10 (a cytokine associated with hepatic inflammation and immunoregulatory pathways in the liver parenchyma) has been proposed as a negative predictor for the response to antiviral therapy in HCV mono-infected (25), and recently in HIV-HCV co-infected patients too (26). It has been shown that both intrahepatic IP-10 mRNA and its mirroring plasma levels are elevated before treatment initiation in chronically HCV infected patients who do not achieve a sustained viral response (27). IP-10 seems to predict the “first phase decline” of HCV-RNA during therapy for all HCV genotypes (28). A high rate of inflammation, and subsequent activation of the endogenous IFN system (29), as well as significant levels of oxidative damage (30), have been proposed as mechanisms for IP-10 actions. Recently, it has been suggested that an important HIV regulatory protein-tat- is capable of inducing IP-10 expression and subsequently enhancing HCV replication in HIV-HCV co-infected individuals (31). In our cohort higher IP-10 levels were directly associated with HCV replication and the progression of liver disease (as shown by high FIB4 index and increased ALT level), as well as with HIV infection markers (HIV viral load and immunosuppression). In accordance with other recent reports (26), a serum IP-10 level of 400 pg/mL can be considered a reliable marker for the evaluation of the severity of liver disease, which may distinguish patients with expected treatment non-response or relapse after antiviral therapy for hepatitis C.To our knowledge, this is the first study providing data on the baseline characteristics of the HIV-HCV co-infected patients in Romania. An important part of these patients have negative baseline viral predictors for both the natural and on-treatment evolution of HCV infection: genotype 1, high baseline HCV viral load and advanced level of fibrosis. Treatment must not be precluded in these patients, and special attention should be paid towards overlapping drug toxicities, as well as to the correctable underlying factors that may alter the response, including patients’ motivation and adherence. The plasma level of IP-10 is a reliable and affordable marker for the progression of liver disease in HIV co-infection.
